# Development and Characterization of a Thermostable Liquid Formulation of Live Newcastle Disease Vaccine

**DOI:** 10.3390/vetsci13040359

**Published:** 2026-04-07

**Authors:** Li Li, Yingying Xu, Junjie Yang, Helong Feng, Hongcai Wang, Zhe Zeng, Lun Yao, Qingping Luo, Guoyuan Wen, Guofu Cheng, Yu Shang

**Affiliations:** 1 College of Veterinary Medicine, Huazhong Agricultural University, Wuhan 430064, China; lili2012@webmail.hzau.edu.cn (L.L.); junjieyangvet@163.com (J.Y.); 2 Key Laboratory of Prevention and Control Agents for Animal Bacteriosis (Ministry of Agriculture and Rural Affairs), Hubei Provincial Key Laboratory of Animal Pathogenic Microbiology, Institute of Animal Sciences and Veterinary Medicine, Hubei Academy of Agricultural Sciences, Wuhan 430064, China; xuyingying@mail.hzau.edu.cn (Y.X.); fenghelong@webmail.hzau.edu.cn (H.F.); whcai2006@163.com (H.W.); zengzhe@hbaas.ac.cn (Z.Z.); yaolun027@126.com (L.Y.); qingping0523@163.com (Q.L.); wgy_524@163.com (G.W.); 3 Hubei Hongshan Laboratory, Wuhan 430064, China

**Keywords:** Newcastle disease virus, liquid vaccine, stabilizer, thermostability, immunogenicity

## Abstract

Vaccination remains the core strategy for the prevention and control of Newcastle disease (ND). The inherent thermosensitivity of traditional Newcastle disease virus (NDV) vaccines imposes major limitations on their transportation, storage, and field application. To address these challenges, a novel liquid, thermostable, live ND vaccine was developed in the present study. Firstly, Tris/HCl buffer at near-neutral pH was identified as the optimal basic buffer system. On this basis, further screening and formulation optimization of vaccine stabilizers were conducted, and NDV strains with excellent thermal stability were used to verify the stability-conferring properties of the developed stabilizer. The results showed that the formulation composed of 0.5% gelatin, 4% trehalose, 0.1% L-glutamic acid, and 0.5% thiourea was confirmed as the optimal stabilizer for ND liquid vaccines. This formulation maintained the stable storage of the tested NDV for 12 months at 4 °C and exhibited promising stability for 30 days at 25 °C, marking a significant advancement toward development thermostable NDV vaccines that are independent of a continuous cold chain. More importantly, the liquid vaccine stored at 4 °C for 12 months still induced high levels of NDV-specific antibodies in specific pathogen-free chicks and provided 100% protective efficacy against challenge with virulent NDV. In conclusion, the liquid vaccine stabilizer developed in this study not only significantly enhanced the thermostability of the vaccine but also effectively maintained its immunogenicity, thereby providing an important theoretical basis for the research and development of liquid ND vaccines.

## Simple Summary

Newcastle disease (ND), caused by Newcastle disease virus (NDV), is a highly contagious disease that seriously endangers the global poultry industry, and vaccination is the core measure for its prevention and control. However, traditional ND vaccines are inherently thermosensitive, which greatly limits their transportation, storage, and field application. To solve this problem, this study developed a novel live thermostable liquid ND vaccine. First, Tris/HCl buffer was selected as the optimal basic buffer system. Then, vaccine stabilizers were further screened and optimized, and NDV strains with excellent thermostability were used to verify the stabilizer effect. The results showed that the optimal stabilizer formulation was composed of 0.5% gelatin, 4% trehalose, 0.1% L-glutamic acid, and 0.5% thiourea. This optimized stabilizer formula effectively enhanced the thermal stability of the liquid vaccine at 25 °C as a preliminary technical improvement, and maintained stable NDV infectivity for 12 consecutive months under 4 °C storage conditions. Notably, the liquid vaccine stored at 4 °C for 12 months still induced high levels of NDV-specific antibodies in specific pathogen-free SPF chicks and provided 100% protection against virulent NDV challenge. In summary, the stabilizer developed in this study effectively improves the thermostability and maintains the immunogenicity of the liquid NDV vaccine, which provides important support for the research and development of liquid ND vaccines and the efficient prevention and control of ND.

## 1. Introduction

Newcastle Disease (ND), a highly contagious and infectious disease caused by Newcastle Disease Virus (NDV), poses a severe threat to the healthy and sustainable development of the global poultry industry [1]. NDV is capable of infecting a wide range of avian species and has been prevalent in more than 100 countries worldwide [2,3,4]. With the rapid development of intensive poultry farming in recent years, it has created favorable conditions for the occurrence of complex multifactorial infections, such as bacterial and viral co-infections [5,6]. Specifically, NDV infection is often accompanied by co-infections with other pathogens or induces secondary infections by other viruses and bacteria, which exacerbates clinical symptoms and increases mortality rates [7,8]. Under this background, the concurrent transmission of multiple pathogens has posed enormous challenges to prevention and control of epidemics, imposing a heavy economic burden on the global poultry industry. Therefore, strengthening the comprehensive prevention and control of ND is crucial for ensuring the healthy development of the poultry industry.

At present, vaccination combined with enhanced biosecurity measures is the most economical and efficient core strategy for the prevention and control of ND [9]. The large-scale application of live vaccines and inactivated vaccines has significantly reduced the incidence and mortality of ND. Inactivated vaccines require individual administration via intramuscular or subcutaneous injection, which is cumbersome, time-consuming, and labor-intensive. Although they can induce high-efficiency and long-lasting humoral immune responses, their ability to induce cellular immunity and mucosal immunity is poor [10]. In contrast, live vaccines have unique advantages in synchronously activating humoral immunity, mucosal immunity and cellular immunity [11]. They can be delivered through diverse routes such as spraying, drinking water and feed mixing [12], which can effectively save labor and epidemic prevention resources. This makes them suitable for the large-scale vaccination needs in the modern intensive poultry industry. However, the aforementioned methods expose vaccines to the external environment for extended periods, which imposes higher requirements on vaccine stability [13]. In addition, traditional live vaccines are highly heat-sensitive, and their immune efficacy is easily impaired by temperature fluctuations during storage and transportation [14], which are highly dependent on a well-established cold chain system. However, during low-temperature storage and transportation, if no appropriate protectants are added, viral proteins may also undergo conformational denaturation, leading to the aggregation of virus particles [15].

Currently, most commercially available live ND vaccines are lyophilized preparations with complex components and cumbersome manufacturing processes. Although the freeze-drying process and the addition of stabilizers can effectively protect the conformational stability of viral proteins, prevent conformational denaturation and particle aggregation, and improve the thermostability of vaccines during long-term storage, these lyophilized vaccines still have obvious limitations. They require reconstitution with special diluents before use, and their efficacy gradually declines with prolonged storage [16]. Moreover, lyophilized vaccines need to be refrigerated at 2~8 °C or lower and rely on a stable cold chain support system during transportation. This significantly increases vaccine costs and imposes a heavy economic burden on routine prevention and control of ND in remote areas and developing countries. Against this background, the research and development of safe, efficient, and thermostable ND vaccines has become increasingly urgent and important.

Thermostable liquid vaccines have opened up new ideas for the development of ND vaccines, owing to their advantages of simple preparation process, convenient inoculation operation, and no requirement for reconstitution. A thermostable recombinant NDV candidate vaccine was designed targeting NDV and infectious bronchitis virus using a modified thermostable NDV as the vector. This vaccine could be stably stored in a liquid environment at 25 °C for 16 days and achieved good immune protection effects. In addition, other researchers [17] have screened stabilizer formulations for liquid vaccines, and these formulations enabled the recombinant NDV expressing the SARS-CoV-2 S protein to maintain the S antigen content throughout the entire six-month test period under storage conditions of 2 °C to 8 °C and 25 °C [18]. This study aims to screen and optimize special stabilizers for NDV, prepare thermostable NDV liquid vaccines, and systematically evaluate their thermal stability and immunogenicity. The findings are expected to provide solid technical support and important theoretical reference for the efficient prevention and control of ND.

## 2. Materials and Methods

### 2.1. Cells, Viruses, and Animals

BHK-21 cells were obtained from our laboratory and cultured in Dulbecco’s modified Eagle’s medium (DMEM) (Gibco, New York, NY, USA) containing 10% fetal bovine serum (FBS) (Gibco) and maintained at 37 °C in an incubator with 5% CO_2_. NDV strains LaSota, TS09-C, and F48E9 were obtained from Hubei Academy of Agricultural Sciences, Wuhan, China. The mutant strains rTS-HN-N3, rTS-HN-UN4, and rTS-HN-PU4 were derived from the thermostable TS09-C strain, while rLS-HN-N10 was derived from the LaSota strain. These four thermostable mutant strains were constructed by introducing targeted point mutations to increase the negative charge of the hemagglutinin-neuraminidase (HN) protein, a key determinant of NDV thermostability, in our previous study [19]. The recombinant modification only enhanced viral thermostability without altering the core biological characteristics, immunogenicity, and safety of the parental strains, making them ideal candidates for evaluating the protective effect of the screened stabilizers and developing thermostable liquid vaccines. All NDV strains were propagated in 9–11-day-old Specific pathogen-free (SPF) chicken embryos. SPF embryonated eggs were purchased from Beijing Boehringer Ingelheim Vital Biotechnology Co., Ltd. (Beijing, China) and hatched in a contained environment at 37.5 °C and 50 to 60% humidity. SPF chicks were hatched from SPF embryonated eggs and raised in biosafety isolators.

### 2.2. Optimization of Stabilizer Buffer System

Four types of dilution buffers, including ultrapure water, 0.9% NaCl, 0.01 M Tris/HCl, and 0.01 M PBS, were used to dilute the allantoic fluid of the TS09-C strain by 10-fold. For thermal stability analysis, viral samples suspended in different dilution buffers were incubated in a 56 °C [20] water bath for 5, 10, 20, 40, 60, and 90 min, respectively. After each time point, samples were immediately transferred to ice for rapid cooling. The hemagglutination (HA) titer of each sample was determined, and all experiments were performed in three biological replicates. The thermal stability of the virus was evaluated by quantifying the reduction rate of HA titer under the above-defined temperature conditions.

Based on the buffer screening results, the pH value of the optimal buffer was adjusted to a series of gradients (5.0, 5.5, 6.0, 6.5, 7.0, 7.5, 8.0, 8.5, and 9.0) using 1 mol KOH or HCl with a pH meter. Subsequently, the TS09-C strain was 10-fold diluted in buffers with different pH values, followed by incubation in 56 °C [20] water bath for 5, 10, 20, 40, 60, and 90 min, respectively. After incubation, the treated samples were placed on ice immediately, and their HA titers measured by the HA assay.

### 2.3. Virus Titration

HA titer determination was performed as described previously [21]. A total of 25 μL of the virus was serially diluted 2-fold in a V-shaped 96-well plate. Freshly prepared 1% chicken red blood cells (RBCs) were added to each well, and the plate was incubated at 37 °C for 15 min. The highest dilution of the virus that caused complete agglutination of chicken RBCs was defined as the HA titer.

The 50% egg infectious dose (EID_50_) of the NDV was determined using 9–11-day-old SPF chicken embryos, as described previously [21]. The virus was serially diluted 10-fold with PBS and 100 μL of each diluted virus sample was inoculated into the allantoic cavity of the SPF chicken embryos. Embryos that died within 24 h post-inoculation (hpi) were discarded. Allantoic fluid was collected at 96 hpi, and the HA titer was detected. The EID_50_ value was calculated using the Reed–Muench method [22].

The 50% tissue culture infectious dose (TCID_50_) assay on BHK-21 cells (a well-standardized and highly sensitive cell line for NDV propagation and titration) was used to determine the NDV viral titer, as described previously [21]. Briefly, the virus was serially diluted 10-fold with DMEM supplemented with 0.2 μg/mL TPCK-Trypsin. A 96-well plate pre-seeded with confluent BHK-21 cells was prepared, and 100 μL of the diluted virus sample was added to each well. The plate was further cultured for 48 h. After incubation, the cells were fixed with 4% paraformaldehyde, permeabilized with 0.5% Triton X-100, and blocked with 3% bovine serum albumin (BSA). NDV-positive serum was used as the primary antibody, and fluorescein isothiocyanate (FITC)-conjugated anti-chicken IgG was used as the secondary antibody. Fluorescent signals were observed and recorded under a fluorescence microscope to determine the infection status (positive or negative). The TCID_50_ value was calculated via the Reed–Muench method [22].

### 2.4. Preparation and Optimization of Liquid Vaccine Stabilizers

Liquid vaccine stabilizers were prepared based on the optimized buffer system (Table 1), and then mixed with the allantoic fluid of the TS09-C strain to formulate liquid vaccines. The prepared liquid vaccines were aliquoted into vials at 1 mL/vial and incubated at 37 °C for 7 days. Viral titers were determined by the TCID_50_ assay to screen for the optimal stabilizer formulation.

According to the stability test results, the concentrations of excipients in the candidate formulations were further optimized to prepare new batches of liquid vaccines. Subsequently, the thermal stability of these optimized liquid vaccines was evaluated under storage at 37 °C to confirm the optimal stabilizer formulation.

The pH value of the optimal formulated stabilizer was adjusted to a range of 6.0–8.5. The pH-adjusted stabilizer was mixed with the allantoic fluid of the TS09-C strain to prepare liquid vaccines, which were then aliquoted into vials and stored at 37 °C for 7 days. Viral titer was determined by the TCID_50_ assay. The optimal pH value of the stabilizer was screened based on the degree of viral titer reduction.

Vaccine stabilizers were prepared using food-grade, industrial-grade, and analytical-grade raw materials, respectively. Each type of stabilizer was mixed with the allantoic fluid of the TS09-C strain to formulate liquid vaccines. After aliquoting into sterile vials, the liquid vaccines were stored at 37 °C for 7 days. Viral titers of all samples were determined via the TCID_50_ assay. The optimal raw material source for the stabilizer was confirmed based on the degree of viral titer reduction, with the raw material corresponding to the minimum titer loss selected as the optimal one.

### 2.5. Selection of Thermostable Vaccine Strains

Candidate NDV strains for thermostable liquid vaccines were screened based on their thermal stability. The allantoic fluid of NDV TS09-C, rTS-HN-N3, rTS-HN-UN4, rTS-HN-PU4, rLS-HN-N10 and commercial vaccine LaSota were individually mixed with the optimally formulated stabilizer to prepare liquid vaccines. After aliquoting, the liquid vaccines were treated in a 56 °C water bath for different durations, and the reduction in HA titer was determined. The vaccines were then stored at 37 °C for 7 days, and viral titer changes were measured by the TCID_50_ assay.

### 2.6. Evaluation of Long-Term Thermal Stability of NDV Liquid Vaccines

Stabilizers were prepared according to the optimal formulation, and their pH was adjusted to the optimal value. The adjusted stabilizers were mixed with stable NDV strains to prepare liquid vaccines, with a final viral titer of 10^7^ EID_50_/mL. The liquid vaccines were aliquoted into vials at 2 mL/vial, with three biological replicates set for each sample. Meanwhile, heat-resistant NDV strains without stabilizer addition served as the control group. All samples were stored at 4 °C or 25 °C. Regular sampling was performed at fixed monthly intervals for a total duration of 12 months under 4 °C storage, and at 10-day intervals within 60 days under 25 °C storage, respectively. The viral titer of each collected sample was determined in parallel via the TCID_50_ assay, as described in Section 2.3. Viral titer loss was calculated at each predetermined sampling time point by subtracting the detected viral titer at the sampling time point from the initial viral titer of the corresponding sample, to quantitatively evaluate the protective effect of the stabilizer on viral activity during long-term storage.

### 2.7. Evaluation of Immunogenicity and Protective Efficacy of Liquid Vaccines

The optimal heat-resistant vaccine candidate strain was mixed with the optimized stabilizer to prepare the liquid vaccine, with a final viral titer adjusted to 10^7.0^ EID_50_/mL. Meanwhile, the vaccine candidate strain without stabilizer addition was mixed with 0.01 M Tris/HCl buffer, and its titer was also adjusted to 10^7.0^ EID_50_/mL. Both preparations were stored at 4 °C for 12 months for subsequent evaluations of thermostability and immunogenicity.

Sixty 7-day-old SPF chicks were randomly divided into 6 groups (Groups 1–6) and immunized via intranasal and intraocular (IN/IO) routes. Chicks in Group 1 and Group 3 were immunized with freshly prepared liquid vaccines with and without vaccine stabilizer at a dose of 10^6.0^ EID_50_ per chick, respectively. Chicks in Group 2 and Group 4 were inoculated with 100 μL liquid vaccines with and without vaccine stabilizer that had been stored at 4 °C for 12 months, respectively. Chicks in Group 5 were immunized with commercial LaSota vaccine at a dose of 10^6.0^ EID_50_ per chick. Group 6 served as the unimmunized control group. Blood samples were collected from the wing veins of SPF chickens weekly for three weeks post-immunization. The levels of NDV-specific hemagglutination inhibition (HI) antibodies in the sera were then determined.

At 21 dpi, chicks in each group were challenged with the virulent NDV strain F48E9 via intramuscular injection at a dose of 10^5.0^ embryo median lethal dose (ELD_50_). Clinical signs of Newcastle disease and chick mortality after challenge were monitored daily for 8 consecutive days.

### 2.8. Hemagglutination Inhibition (HI) Assay

NDV-specific antibodies in serum were detected by the HI assay, as described previously [23]. Briefly, serum samples were serially diluted 2-fold in a V-shaped 96-well plate. An equal volume of 4 hemagglutinating units (HAU) of NDV antigen was added to each well, and the plate was gently mixed and incubated at room temperature for 15 min. Subsequently, freshly prepared 1% RBCs were added to each well, followed by gentle mixing again. After standing at room temperature for another 15 min, the highest dilution of serum that completely inhibited the HA activity of NDV antigen was defined as the HI titer of the sample.

### 2.9. Statistical Analysis

All data were expressed as the mean ± standard deviation (SD) for each group. One-way analysis of variance (ANOVA) or two-way ANOVA was used for data analyses. Statistical significance was defined as follows: *, *p* < 0.05; **, *p* < 0.01; and ***, *p* < 0.001.

## 3. Results

### 3.1. Optimal Buffer System for NDV

**Figure 1:**
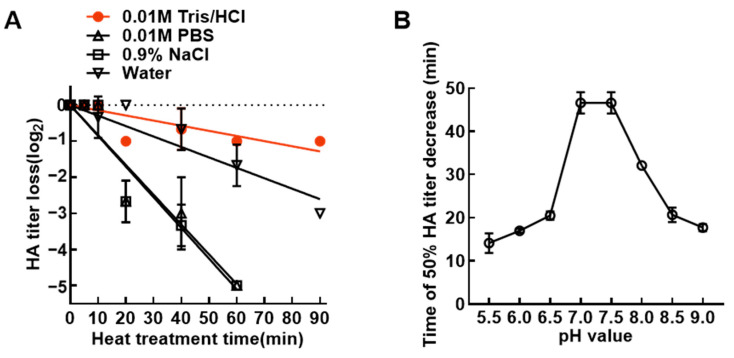
Screening of the optimal buffer system and pH optimization for NDV vaccines. (**A**) Viral allantoic fluid was 10-fold diluted with different buffer systems (pH 7.0), including 0.01 M Tris/HCl, 0.01 M PBS, 0.9% NaCl, and water. Viral samples suspended in different dilution buffers were incubated in a 56 °C water bath for 5, 10, 20, 40, 60, and 90 min. The HA titer of TS09-C strain was detected, and all samples were subjected to three biological replicates. (**B**) For pH optimization, the allantoic fluid of TS09-C was 10-fold diluted with 0.01 M Tris/HCl buffers of different pH values. After heat treatment at 56 °C for various time periods, the curve of HA titer changes with heat treatment time was plotted, and the time required for a 50% decrease in HA titer (T_50_) was calculated.

The buffer systems were screened for their ability to maintain the thermal stability of NDV. As shown in Figure 1A, the virus in 0.01 M Tris/HCl exhibited the highest stability, of which the HA titer decreased by approximately 1.0 log_2_ after heat treatment at 56 °C for 90 min. In contrast, the virus showed moderate thermal stability in water, while the poorest stability was observed in 0.9% NaCl and 0.01 M PBS buffers. These results demonstrate that the buffer type has a significant impact on NDV stability, and 0.01 M Tris/HCl was identified as the optimal buffer for subsequent NDV stabilizer screening experiments.

Further optimization of pH was performed for the selected buffer system. The results indicated that pH 7.0 and 7.5 in 0.01 M Tris/HCl were favorable for maintaining the thermal stability of TS09-C strain. Under heat stress at 56 °C, the time required for a 50% reduction in HA titer (T_50_) of the TS09-C strain in these pH conditions was 47 min (Figure 1B). The T_50_ values varied significantly across different pH gradients, confirming that the pH of the buffer system was a critical factor affecting NDV thermal stability. Collectively, these data suggested that Tris/HCl buffer with a near-neutral pH (7.0–7.5) is conducive to maintaining the thermal stability of NDV.

### 3.2. Screening and Optimization of Liquid NDV Vaccine Stabilizer Formulations

**Figure 2:**
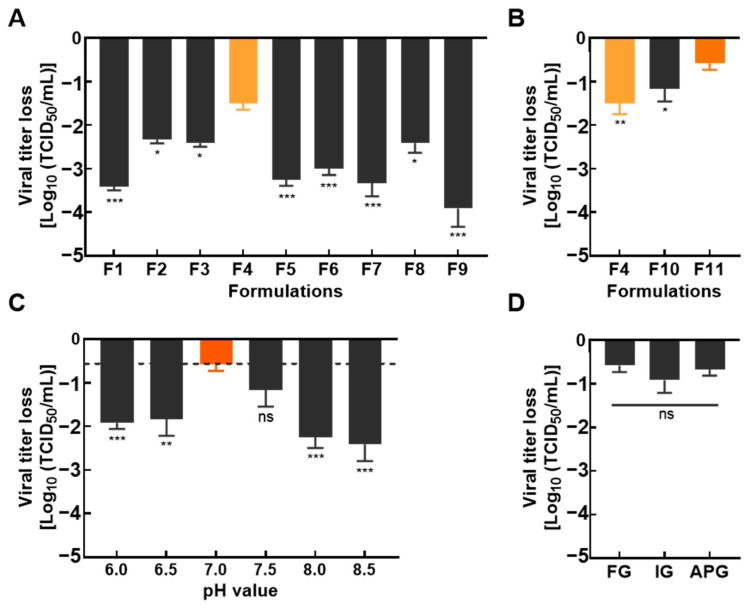
Screening and optimization of NDV vaccine stabilizer formulations. (**A**) Liquid vaccines were prepared by mixing TS09-C strain with stabilizers of different formulations. After incubation at 37 °C for 7 days, the virus was serially diluted 10-fold, inoculated into BHK-21 cells, and the TCID_50_ of TS09-C was determined to evaluate the titer reduction. (**B**) For further optimization of stabilizer F4 formulation, the concentration of gelatin was adjusted to 1.5% and 0.5%. Liquid vaccines were prepared by mixing the optimized formulations with the TS09-C strain. After incubation at 37 °C for 7 days, the liquid vaccines were serially diluted 10-fold, inoculated into BHK-21 cells, and the viral titer was detected to assess the titer reduction. (**C**) The pH of the optimal stabilizer formulation was adjusted to 6.0, 6.5, 7.0, 8.0, and 8.5, respectively. Liquid vaccines were prepared by mixing each pH-adjusted stabilizer with the TS09-C strain, aliquoted, and stored at 37 °C for 7 days. The viral titer was measured to evaluate the reduction of TCID_50_ titer. (**D**) Using 0.01 M Tris/HCl as the buffer system, F11 vaccine stabilizer was prepared with food-grade (FG), industrial-grade (IG), and analytical-grade (APG) raw materials, respectively, and the pH was adjusted to 7.0. Liquid vaccines were prepared by mixing each stabilizer with the TS09-C strain, stored at 37 °C for 7 days, and the TCID_50_ titer was determined. For data analysis, all groups were compared with the experimental group with the least titer reduction for difference analysis using one-way ANOVA. Significance levels are indicated as: *, *p* < 0.05; **, *p* < 0.01; ***, *p* < 0.001.

To screen optimal stabilizer formulations for NDV liquid vaccines, various stabilizer formulations were prepared according to Table 1. Each formulation was mixed with the TS09-C strain to prepare liquid vaccines, and viral titer reduction was evaluated after storage at 37 °C for 7 days. As shown in Figure 2A, the vaccine without any stabilizer (F1) exhibited a significant titer reduction, with a decrease of approximately 3.5 log_10_TCID_50_. In contrast, groups supplemented with gelatin (Formulation F2, F3, and F4) showed a less significant decrease in viral titer. The formulation F5 supplemented with both thiourea and Vitamin C failed to effectively inhibit the viral titer attenuation. Among all tested formulations, Formulation F4 (containing 3% gelatin, 4% trehalose, 0.1% L-glutamic acid, and 0.5% thiourea) showed the most significant improvement in the thermal stability of the liquid vaccine, with its TCID_50_ titer decreased by only 1.5 log_10_TCID_50_ after storage at 37 °C for 7 days. For Formulation F8, where gelatin in F4 was replaced with skim milk, the thermal stability of the vaccine was also improved; however, further optimization experiments for this formulation was not performed due to the high cost of skim milk.

Owing to the poor fluidity of the vaccine stored at 4 °C, the working concentration of gelatin in F4 was further optimized. As shown in Figure 2B, the virus in Formulation F11 (containing 0.5% gelatin) exhibited the best thermal stability, with a titer reduction of approximately 0.5 log_10_TCID_50_ after 7 days of storage at 37 °C. Importantly, the liquid vaccine prepared with this gelatin concentration maintained good fluidity without jelly-like precipitation during storage at 4 °C.

The addition of excipients may alter the pH of stabilizers. Therefore, the optimal pH of the stabilizer was further optimized. The results showed significant differences in thermal stability among vaccines with different pH values. The vaccine exhibited the highest thermal stability at pH 7.0, with a titer reduction of approximately 0.5 log_10_TCID_50_ after 7 days of storage at 37 °C (Figure 2C). Collectively, these results confirmed that Formulation F11 at pH 7.0 is the optimal stabilizer for NDV liquid vaccines.

Based on the development results of stabilizer Formulation F11, three liquid vaccines using excipients of different grades; namely, analytical grade (APG), food grade (FG), and industrial grade (IG). After 7 days of storage at 37 °C, no significant difference was observed in the degree of viral titer reduction among the three liquid vaccines (Figure 2D). This finding demonstrated that the excipient source had no effect on stabilizer performance.

### 3.3. Screening of Thermostable Strains of NDV

**Figure 3:**
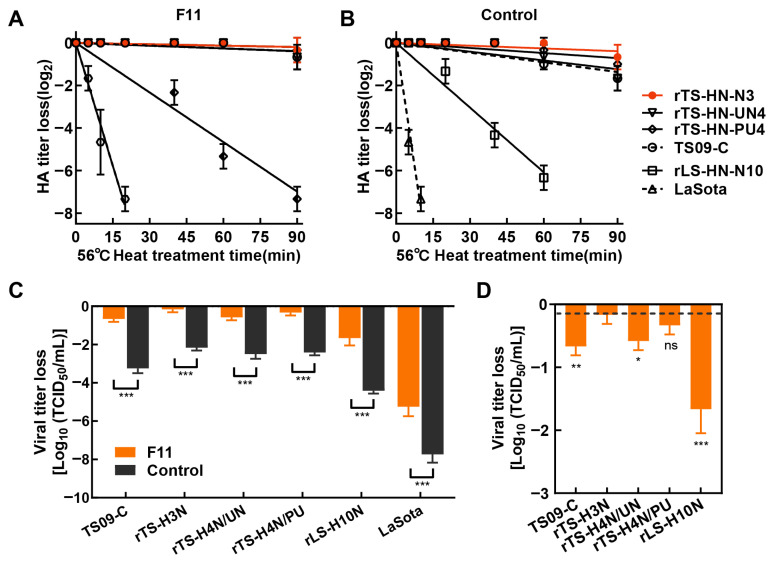
Thermal stability of liquid vaccines prepared with different strains. Liquid vaccines were individually formulated by mixing each strain (TS09-C, rTS-HN-N3, rTS-HN-UN4, rTS-HN-PU4, rLS-HN-N10, and LaSota) with (**A**) or without (**B**) the optimal stabilizer formulation F11. The liquid vaccines were aliquoted and treated in a 56 °C water bath, and the reduction in viral HA titer was determined by the HA assay. (**C**) The vaccines with or without formulation F11 were then stored at 37 °C for 7 days, and viral titer were measured by the TCID_50_ assay. Two-way ANOVA was performed using GraphPad Prism software 10, with significance levels indicated as: *, *p* < 0.05; **, *p* < 0.01; ***, *p* < 0.001. (**D**) Comparison of thermal stability of all heat-resistant liquid vaccines with stabilizers. For data analysis, all groups were compared with the experimental group exhibiting the least titer reduction, and statistical significance was determined using one-way analysis of variance (ANOVA). Significance levels are indicated as follows: *, *p* < 0.05; **, *p* < 0.01; ***, *p* < 0.001.

Subsequently, thermostable NDV strains suitable for liquid vaccine production were further screened. Candidate strains preserved in our laboratory were mixed with stabilizer F11 to prepare liquid vaccines. The thermal stability of these vaccines was evaluated under 56 °C and 37 °C, respectively. As shown in Figure 3A,B, the addition of vaccine stabilizer significantly improved the thermal stability of HA activity in all tested viruses. Among these, the liquid vaccine prepared with rTS-HN-N3 exhibited superior thermal stability compared to other strains, following treatment at 56 °C for 90 min, its HA titer remained almost unchanged. The results (Figure 3C) indicated that vaccine stabilizer also significantly enhanced the thermal stability of viral infectivity, including that of the non-heat-resistant LaSota strain. Notably, the vaccine based on rTS-HN-N3 strain showed the best thermal stability with a titer loss of only 0.2 log_10_TCID_50_. Collectively, these results confirmed that liquid vaccine stabilizer F11 can generally improve the thermal stability of NDV, and the rTS-HN-N3 strain was a promising candidate for the development of heat-resistant NDV liquid vaccines.

### 3.4. Evaluation of Thermal Stability of Liquid Vaccines

**Figure 4:**
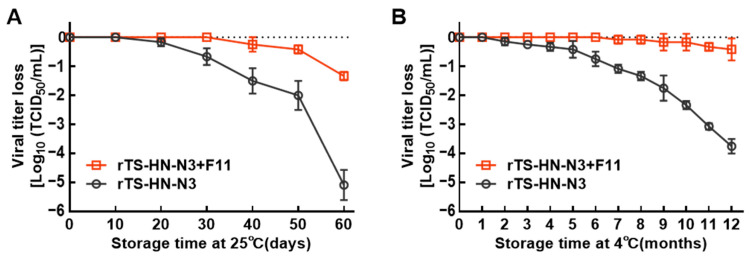
Thermal inactivation kinetics of liquid vaccines at 4 °C and 25 °C. The NDV liquid vaccine was formulated with the rTS-HN-N3 strain and stabilizer formulation F11 (designated as rTS-HN-N3 + F11), and stabilizer-free rTS-HN-N3 was set as the control group. (**A**) The liquid vaccines were stored at 25 °C for 60 days, with samples were collected every 10 days in triplicate. (**B**) The liquid vaccines were stored at 4 °C for 12 months, with samples collected monthly in triplicate. Viral titers were determined via the TCID_50_ assay.

The thermal stability of NDV liquid vaccine prepared with the rTS-HN-N3 strain and stabilizer F11 (rTS-HN-N3 + F11) was evaluated at 25 °C and 4 °C. As shown in Figure 4A, the vaccine rTS-HN-N3 + F11 maintained stability for 30 days at 25 °C, with a titer loss of 1.3 log_10_TCID_50_ at 60 days. In contrast, the rTS-HN-N3 without stabilizer exhibited a gradual titer decline starting at 20 days, with a total reduction 3.5 log_10_TCID_50_ by day 60. At 4 °C, the titer of rTS-HN-N3 + F11 retained unchanged for 6 months, and only a 0.25 log_10_TCID_50_ titer reduction was observed after 12 months of storage. In contrast, the stabilizer-free rTS-HN-N3 vaccine showed a titer decline starting from month 2 at 4 °C, with reductions of 0.75 log_10_TCID_50_ at 6 months and 3.75 log_10_TCID_50_ at 12 months (Figure 4). Collectively, these results demonstrate that the addition of stabilizer F11 significantly improved the thermal stability of the liquid vaccine, which could be stably stored for at least 12 months at 4 °C and 1 month at 25 °C.

### 3.5. Evaluation of Immunogenicity and Protective Efficacy of the Liquid Vaccine

**Figure 5:**
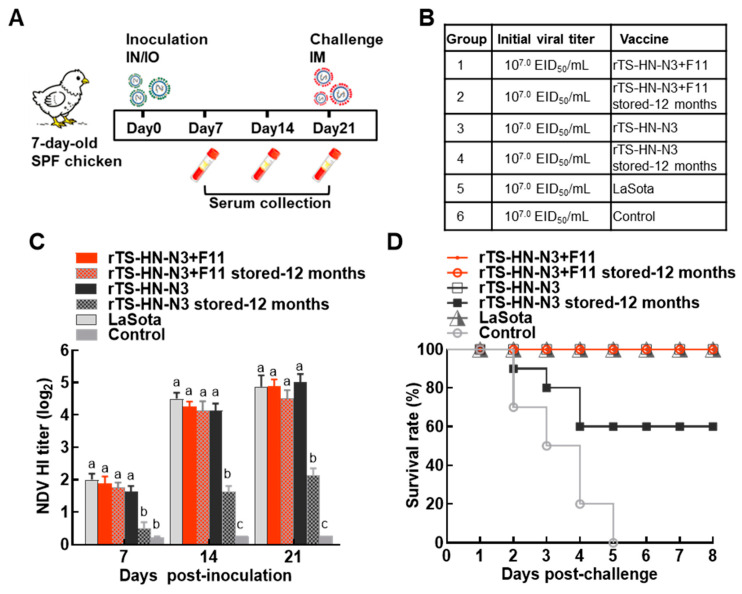
Evaluation of immunogenicity and protective efficacy of the heat-resistant liquid vaccine. (**A**) Experimental design and grouping. Sixty 7-day-old SPF chickens were randomly divided into 6 groups and immunized via intranasal and intraocular (IN/IO) at a dose of 100 μL per chicken. Blood samples were collected from the wing vein every 7 days, and serum NDV-specific antibodies were detected via HI assay. At 21 dpi, all chickens were challenged with the virulent F48E9 strain via intramuscular injection (IM), and mortality was monitored and recorded daily. (**B**) Immunization groups and types of immunized vaccines. (**C**) NDV-specific antibody levels detected by the HI assay. Statistical significance of differences was analyzed by two-way ANOVA. Values with different lowercase letters (a, b, c, etc.) indicate significant differences (*p* < 0.05), while the same lowercase letters indicate no significant difference. (**D**) Survival rate of chickens in each group following challenge with the virulent NDV F48E9 strain.

The immunogenicity of the liquid vaccine was evaluated in 7-day-old SPF chickens. NDV-specific antibody titers were presented in Figure 5A,B. The addition of vaccine stabilizer did not interfere with the immunogenicity of the vaccine. Specifically, no significant differences in antibody levels were detected between chickens immunized with rTS-HN-N3 + F11 and those immunized with rTS-HN-N3 (without stabilizer) at 7, 14, and 21 days post-inoculation (dpi). At 21 dpi, the NDV-specific antibody titer was approximately 2^5^, which was comparable to that in chickens immunized with the commercial lyophilized LaSota vaccine. Notably, rTS-HN-N3 supplemented with stabilizer still induced high levels of NDV-specific antibodies (HI titer: 2^4.5^) following storage at 4 °C for 12 months. In contrast, the antibody level induced by rTS-HN-N3 without stabilizer was significantly lower than that in the fresh vaccine-immunized group (Figure 5C).

At 21 dpi, all immunized and control chickens were challenged with virulent NDV F48E9 strain to assess the protective efficacy of the liquid vaccine. All chickens in the unimmunized control group died within 5 days post-challenge (dpc). During the 8-day observation period post-challenge, 100% of chickens in the fresh liquid vaccine group and commercial LaSota vaccine group survived without manifesting obvious clinical symptoms. Importantly, the vaccine containing stabilizer still conferred 100% protective efficacy against virulent challenge even after storage at 4 °C for 12 months. In contrast, the survival rate of chickens immunized with rTS-HN-N3 without stabilizer was only 60% (Figure 5D).

## 4. Discussion

Newcastle disease virus (NDV) poses a persistent threat to the global poultry industry, with vaccination representing the cornerstone of prevention and control. In addition to its use as a conventional vaccine, NDV has emerged as a versatile viral vector owing to its favorable properties, including strong immunogenicity, low pathogenicity in mammals and most avian species, and high efficiency in expressing heterologous antigens from diverse pathogens [24]. However, the widespread application of NDV vaccines is severely hindered by two major limitations: the inherent thermosensitivity of most NDV strains and the disadvantages of conventional lyophilized formulations, which involve complex manufacturing processes and high costs [25]. To overcome these obstacles, the present study developed a lyophilization-free liquid NDV vaccine characterized by long-term stability. This approach not only provides an alternative strategy for ND control, but also establishes a technical platform for the development of liquid NDV-vectored vaccines

Temperature is a major factor triggering viral inactivation and buffer systems are known to modulate thermal stability by shaping the physicochemical microenvironment. Enveloped viruses are particularly susceptible to pH shifts, as extreme pH can induce conformational alterations in surface proteins, leading to aggregation and loss of infectivity [26,27]. In this study, screening of different buffers demonstrated that 0.01 M Tris/HCl buffer at pH 7.0 or 7.5 was superior to PBS, 0.9% saline, and water in slowing viral titer decay under thermal stress. These results are consistent with previous reports indicating that near-neutral or weakly acidic pH maximizes viral stability, whereas extreme acidity or alkalinity accelerates inactivation of enveloped viruses [28,29], highlighting the essential role of appropriate buffer systems in maintaining NDV infectivity.

Nonetheless, a single buffer system is insufficient to achieve long-term stability in liquid formulations, and studies on stabilizers for NDV liquid vaccines remain limited, with protective efficacy varying substantially among virus. Here, multiple excipients were screened, and a composite stabilizer (F11) consisting of 0.5% gelatin, 4% trehalose, 0.5% thiourea, and 0.1% L-glutamate was identified to significantly enhance NDV thermal stability.

Gelatin, a classical vaccine stabilizer, forms a semi-solid matrix that inhibits viral aggregation, phase transition, and surface degradation [18]. Synergistic stabilization by trehalose and gelatin has been documented in liquid classical swine fever vaccines stored at 37 °C [30], supporting the present observations. Trehalose functions as a chemical chaperone, forming an amorphous sugar glass that immobilizes viral proteins and envelopes while replacing structural water, thereby improving the thermal stability and immunogenicity of the vaccine [31,32]. Moreover, trehalose offers advantages over other disaccharides, it forms fewer hydrogen bonds with protein surfaces than sucrose, allowing more effective preservation of protein conformations [33]. L-glutamate act as a compatible osmolyte that stabilizes viral protein structures and reinforces the protective effects of sugars [34]. Thiourea, a redox modulator previously used in thermostable lyophilized vaccines, further reduces oxidative damage in trehalose-containing matrices [35]. Notably, the F11 formulation exerted broad-spectrum protection, improving stability in both thermotolerant strains and thermosensitive variants such as LaSota, thus expanding its applicability across multiple NDV strains.

Cost-effectiveness is a critical for the translation of vaccine technologies into field applications. Comparative analysis showed that stabilizers prepared with food-grade, industrial-grade, and analytical-grade excipients all effectively preserved NDV liquid vaccines stability at 37 °C, with viral titer reduction below 1.0 log_10_TCID_50_ after 7 days. Given the lower cost of food-grade raw materials, they represent an optimal choice for large-scale production. Furthermore, removal of the lyophilization step reduces production costs, alleviating a key economic barrier in veterinary vaccine development.

Thermal stability of live vaccines arises from the combined effects of strain-intrinsic thermotolerance and extrinsic formulation protection. In previous study, the thermotolerant attenuated strain TS09-C was obtained by serial screening [36], and further analysis revealed that its thermotolerance was positively correlated with the surface negative charge of the HN protein. Based on this mechanism, a panel of thermotolerant recombinant strains was constructed [19]. Among these, the rTS-HNN3 strain showed the highest thermal stability, with its HA titer decreasing by only approximately 0.5 log_2_ after treatment at 56 °C for 90 min. These findings imply that the intrinsic t thermotolerance of genetically modified strains may approach a biological ceiling, suggesting that formulation optimization represents a more feasible strategy for achieving further improvements in vaccine stability. Notably, although rTS-HN-N3 alone showed significant titer decline after 3 months of storage at 4 °C, the addition of F11 stabilizer maintained stable viral titer for at least 12 months. These results demonstrate synergistic enhancement of stability through intrinsic strain resistance and extrinsic formulation protection. Additionally, the Tris/HCl buffer and major components of the F11 stabilizer are low-ionic ingredients. Combined with a suitable pH, these components do not significantly alter the surface negative charge of virions, thereby preventing protein denaturation and viral aggregation and further supporting the thermal stability of NDV strains.

Immunogenicity and protective efficacy are the core criteria for vaccine quality. Our results confirmed that the liquid vaccine formulated with F11 elicited robust humoral responses (HI titer ≥ 4 log_2_), and conferred 100% protection against lethal NDV challenge, even following 12 months of storage at 4 °C. In contrast, vaccines without the F11 stabilizer showed drastically reduced antibody levels and diminished protective efficacy. This validated that our thermostable liquid vaccine elicits sufficient immune responses in chickens even after long-term storage. Furthermore, the rTS-HN-N3, derived from TS09-C via three amino acid mutations in the HN protein, inherits TS09-C’s potential as a viral vector. TS09-C has previously been successfully engineered to express heterologous antigens [23,37,38], conferring effective protection against the corresponding pathogens. This strongly suggests that rTS-HN-N3 could serve as a vector for developing multivalent thermotolerant liquid vaccines, opening avenues for combating multiple pathogens with a single formulation.

Despite these advances, this study has limitations. While the vaccine demonstrated reliable long-term stability at 4 °C for up to 12 months, its performance at 25 °C, while satisfactory for short-term storage (30 days), still leaves room for further improvement. This achievement marks an important milestone in overcoming the cold chain challenge for NDV vaccines, and future optimizations will aim to extend its ambient temperature stability further.

In conclusion, the integration of the thermotolerant rTS-HN-N3 strain with the optimized F11 formulation (pH 7.0) yielded a thermostable live liquid NDV vaccine. This strategy provides a theoretical framework for the rational design of next-generation cold chain-independent veterinary vaccines.

## Figures and Tables

**Table 1:** Formulations of vaccine stabilizer.

Formulation	Gelation	Skim Milk	Trehalose	L-glutamate	Vitamin C	thiourea	Tris/HCl
	Final Concentration (*w*/*v*%)						
F1	-	-	-	-	-	-	+
F2	3	-	-	-	-	-	+
F3	3	-	4	0.1	0.1	-	+
F4	3	-	4	0.1	-	0.5	+
F5	3	-	4	0.1	0.1	0.5	+
F6	-	5	-	-	-	-	+
F7	-	5	4	0.1	0.1	-	+
F8	-	5	4	0.1	-	0.5	+
F9	-	5	4	0.1	0.1	0.5	+
F10	1.5	-	4	0.1	-	0.5	+
F11	0.5	-	4	0.1	-	0.5	+

Note: 0.01 M Tris/HCl, with a targeted pH of 7.0. note: “-” indicates that the formulation did not contain the corresponding excipient; “+” indicates that each formulation was dissolved in 0.01 M Tris/HCl.

## Data Availability

The data presented in this study are available on request from the corresponding authors due to privacy or ethical restrictions.
